# Combination of high resolution MRI with 3D-printed needle guides for ex vivo myocardial biopsies

**DOI:** 10.1038/s41598-023-50943-2

**Published:** 2024-01-05

**Authors:** Simon Reiss, Julien Thielmann, Johannes Fischer, Thomas Lottner, Alexander Maier, Dirk Westermann, Constantin von zur Mühlen, Timo Heidt, Michael Bock

**Affiliations:** 1 Division of Medical Physics, Department of Diagnostic and Interventional Radiology, Faculty of Medicine, University Medical Center Freiburg, University of Freiburg, Killianstr. 5a, 79106 Freiburg, Germany; 2 Department of Cardiology and Angiology, Faculty of Medicine, University Heart Center Freiburg-Bad Krozingen, University of Freiburg, Freiburg, Germany

**Keywords:** Preclinical research, Cardiovascular diseases, Biomedical engineering

## Abstract

Magnetic resonance imaging (MRI) provides a multitude of techniques to detect and characterize myocardial infarction. To correlate MRI findings with histology, in most cases terminal animal studies are performed; however, precise extraction and spatial correlation of myocardial tissue samples to MRI image data is difficult. In this proof of concept study, we present a 3D-printing technique to facilitate the extraction of tissue samples from myocardial regions. Initially, seven pig hearts embedded in formaldehyde were imaged on a clinical 3 T system to define biopsy targets on high resolution ex vivo images. Magnitude images and R2*-maps acquired with a 3D multi-echo gradient echo sequence and 0.58 mm isotropic resolution were used to create digital models of the cardiac anatomy. Biopsy guides were 3D-printed to steer the extraction of myocardial samples. In total, 61 tissue samples were extracted with an average offset of the tissue sample location from the target location of 0.59 ± 0.36 mm. This offset was not dependent on the distance of the target point to the epicardial surface. Myocardial tissue could be extracted from all samples. The presented method enables extraction of myocardial tissue samples that are selected by ex vivo MRI with submillimeter precision.

## Introduction

Cardiovascular magnetic resonance (CMR) provides a multitude of imaging techniques to detect and characterize myocardial infarction (MI). In particular, relaxometry and diffusion tensor imaging are increasingly used to gain insight into the morphology and dynamics of MI^1,2^. Therefore, CMR findings are often correlated to histology after experimentally induced MI in terminal animal studies. In these studies gross histology slices are cut manually from a specific myocardial region^3–6^. As the formalin preservation and cutting procedure can lead to substantial tissue deformation, spatial correlation between the extracted tissue and the ex vivo MR images can be difficult. Furthermore, ex vivo Magnetic Resonance Imaging (MRI) allows for a resolution of 0.5 mm or less at clinical systems which sets a lower spatial resolution limit for any correlation study. However, a reproducible and precise spatial correlation is of particular importance to correctly transfer histology information into clinical imaging biomarkers.

3D printing is a widely available and cost-efficient technique to produce individualized guides and holders. It has previously been used to specify cutting planes for histologic section of rodent brains, allowing for easy co-registration with volumetric MRI data^7^ as well as in vivo targeted biopsy and drug delivery in canine brains^8^. Here, a novel technique is introduced that uses individualized 3D-printed cardiac holders with needle guides to enable precise extraction of tissue samples from myocardial regions of interest that are pre-defined on ex vivo MRI.

## Materials and methods

### Explanted pig hearts

**Figure 1:**
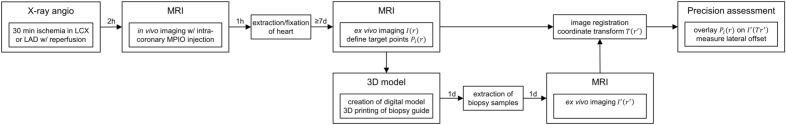
Flow chart showing the key experiments and steps of this study.

Approval of all ethical and experimental procedures and protocols was granted by the Local Ethics Committee of Freiburg University and the Regional Council of Freiburg, Baden-Wuerttemberg, Germany, under Approval Nos. G-15/156, G-16/78, G-19/70, and G-21/008. Experiments were conducted in accordance with FELASA, GV-SOLAS guidelines for animal welfare and reported in accordance with ARRIVE guidelines. German landrace pigs were purchased from Franz und Thomas Lais GbR, Hartheim-Bremgarten 7–10 days prior the experiments and were kept at the large animal facility of the Center for Experimantal Models and Transgenic Service (CEMT), University of Freiburg under the care of experienced staff veterinarians, received water ad libitum and were fed twice a day. An overview of the experiments and key steps performed in this study is shown in Fig. 1. In total, 7 resected pig hearts were used after the animals underwent an interventional CMR study^9–11^ at a clinical 3 Tesla MRI system (PrismaFit, Siemens). The pigs underwent myocardial ischemia of 30 min followed by reperfusion. During the study, the left coronary artery was intubated using an active coronary guiding catheter. A target-selective microparticle of iron oxide (MPIO) based contrast agent was injected through this guiding catheter to assess the potential binding to regions with ischemic injury followed by reperfusion^12–14^. Within one hour after the intracoronary injection of the contrast agent, animals were euthanized by intravenous injection of potassium chloride (2 mmol/kg bw). The hearts were excised, flushed with saline solution and stored in a cylindrical plastic container (diameter: 10 cm, height: 12 cm) filled with 4% paraformaldehyde (PFA). The hearts were stored at room temperature for at least 7 days to allow for the fixative to diffuse through the whole tissue and to create a homogeneous imaging contrast.

### MR Imaging sequence and postprocessing: from imaging to 3D-printing

**Figure 2:**
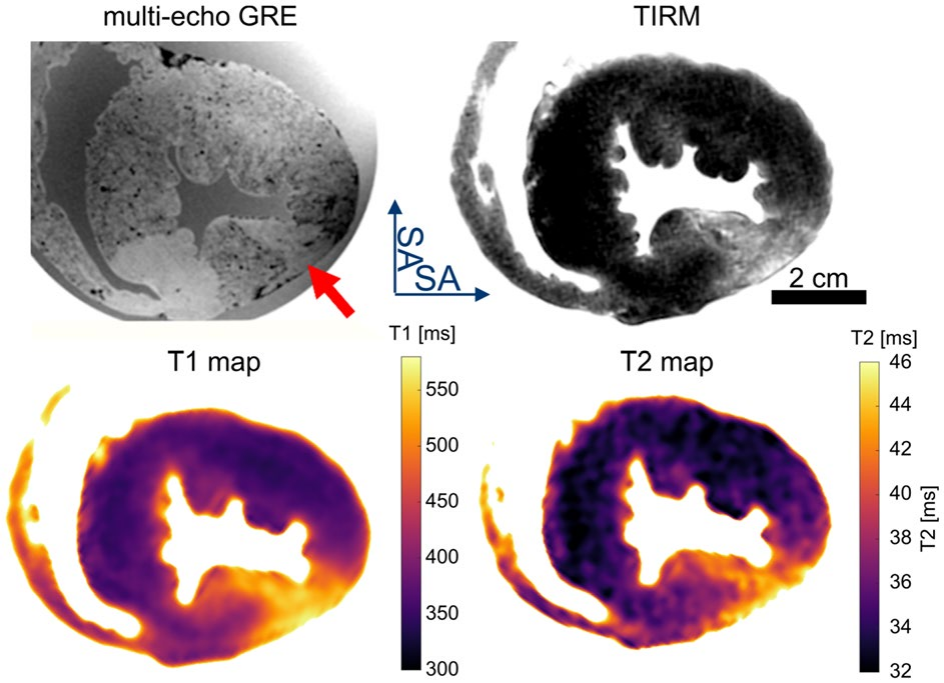
Ex vivo MRI of an extracted pig heart after experimentally induced myocardial infarction with 30 min ischemia followed by 4 h of reperfusion before sacrificing the animals. MPIOs were injected intracoronary 2 h after induction of myocardial infarction. Images were acquired 7 days after fixation with 4% paraformaldehyde. In this example, a microvascular occlusion is seen with no perfusion of MPIOs and increased T1 and T2 values (red arrow).

Ex vivo imaging was performed at the same 3 T MRI system as the in vivo experiments. The hearts were positioned in the plastic container containing the fixative inside a 64 channel head/neck receive only coil. The body coil of the scanner was used for RF transmission. The ex vivo imaging (Fig. 2) included a 3D multi-echo spoiled gradient echo sequence (GRE) with 0.58 mm isotropic resolution covering the whole heart (TR = 23 ms, TE = [3.4, 9.8, 17.1] ms, α = 12°, BW = 260 Hz/px, FoV = 129 × 129 × 84 mm^3^, matrix = 224 × 224 × 144, averages: 2) to quantify the presence of the contrast agent by its T2*-shortening effect. Maps of R2* were calculated from the magnitude images of the three echoes by a voxel-wise linear fit to the logarithmic signal intensity. Both the magnitude images and R2* maps were used for visualizing the MPIO contrast agent distribution and to create an individual digital 3D model of each heart to plan the extraction of the tissue samples.

**Figure 3:**
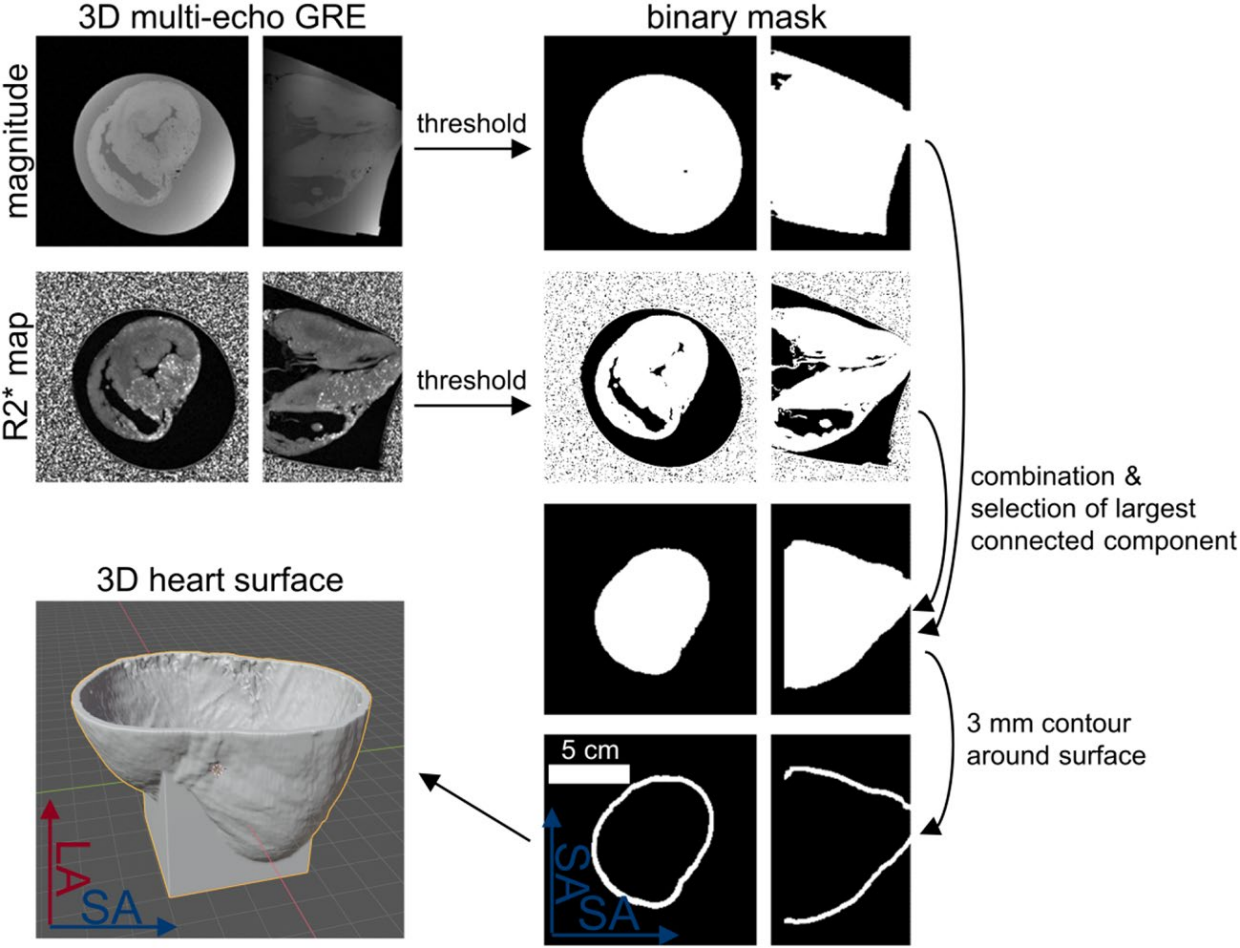
Post-processing of the high resolution multi-echo images to create a surface model for 3D-printing that holds the corresponding heart.

The post-processing to obtain the models was done in Matlab version R2021a (The MathWorks Inc., USA); an overview of the pipeline is illustrated in Fig. 3. First, the magnitude images of the first echo were used to create a binary mask of the hyperintense fixative and the hypointense heart. Therefore, a signal intensity (SI) threshold was used to suppress the region outside the container, i.e. air. This is due to the noise present in this area leading to unreliable R2* values and thus affecting the automatic selection of the myocardium based on R2* values in the next step (see below). The magnitude threshold was set manually for each heart based on a histogram of the signal intensities of all voxels (Supplementary Fig. S1a). The value for the threshold was chosen to exclude the noise peak at low signal intensities. In addition, a region growing algorithm was used with a seed point positioned in the center of the container to select a mask with a single connected component. The myocardium was segmented from the R2* images using a threshold value. As the R2* values of the fixative and the myocardium are (2.98 ± 0.53) s^−1^ and (31.2 ± 3.5) s^−1^, respectively, the threshold was set to 10 s^−1^ (Supplementary Fig. S1b). After taking the intersection of both the magnitude and R2* masks, the largest connected component of the mask was selected (MatLab function *bwconncomp*). Thus, residual non-zero entries of the binary mask outside the myocardium were suppressed that may arise from noise. In addition, the ventricles and atria were added to the binary mask such that the mask has a single boundary surface, which represents the epicardial surface. A 3 mm-thick shell was then created around this surface, which is used to hold the heart when tissue samples are extracted. The surface covered the ventricles from apex to the level of the valves where the diameter of the heart is maximal to facilitate insertion of the heart. Thus, target locations can be chosen covering the whole ventricle.

### Defining the target location

**Figure 4:**
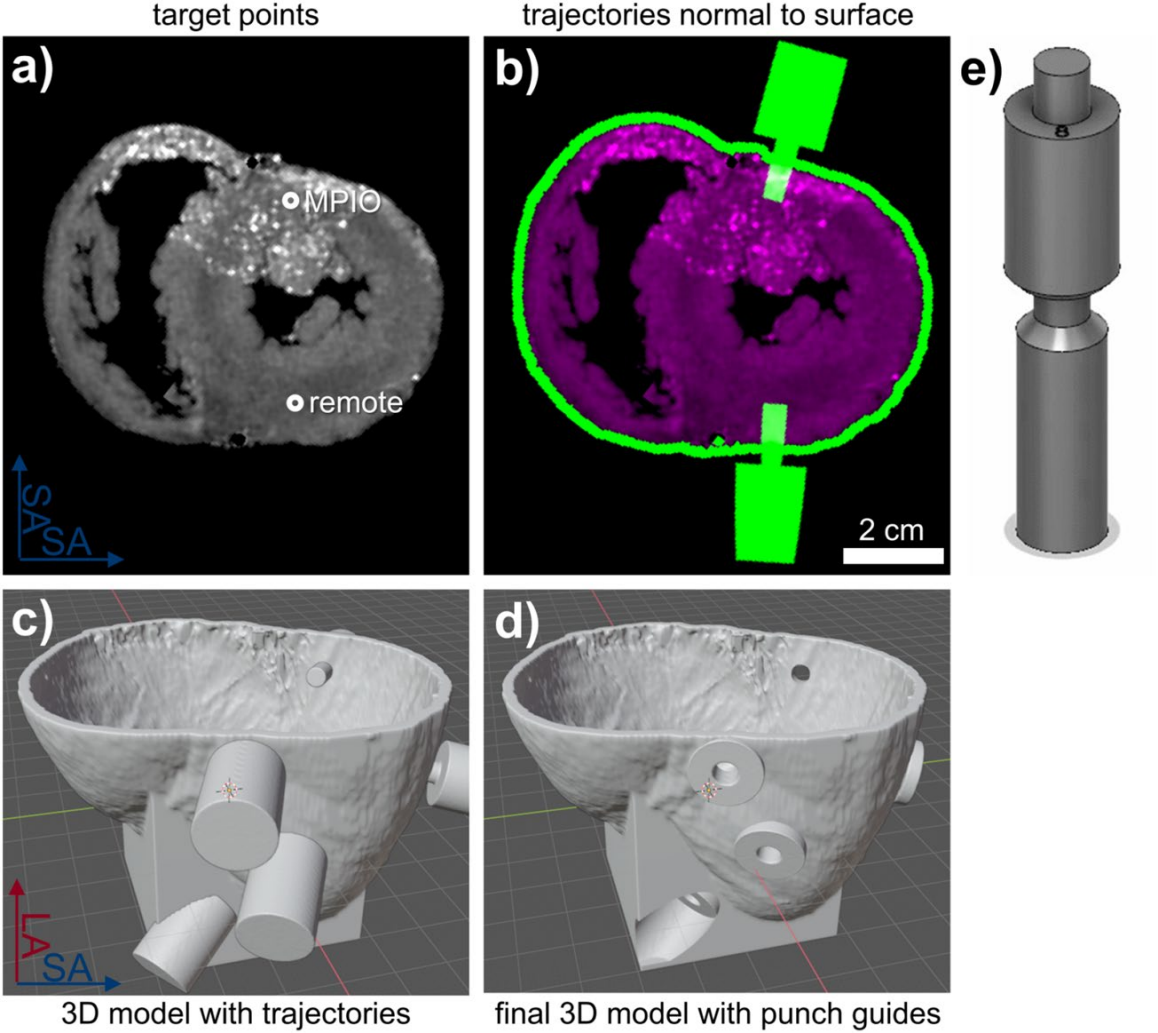
Planning of the trajectories for the biopsy punch. (**a**) Target points in both remote area and area with high MPIO density are manually selected using the R2* maps. (**b**) The trajectories normal to the heart surface are automatically calculated. (**c**) The trajectories to the target points are converted to 3D models of the biopsy punch. (**d**) The final 3D model contains the heart surface and cylindrical guides to each target point for the biopsy punch. (**e**) Digital model of the punch with exchangeable spacers to define the insertion depth of the punch.

The target locations were defined manually in the magnitude images and the R2* maps. Here, multiple targets were selected for each heart from both remote areas where no MPIO induced signal is observed and areas with intense MPIO induced signal (Fig. 4a). Targeting trajectories are then calculated normal to the epicardial surface (Fig. 4b). Around these trajectories, a 5 mm wide and 5 mm long cylinder is modeled (Fig. 4c) to add a hollow guide for a biopsy punch to the 3D model of the epicardial surface of the heart (Fig. 4d).

### MRI based 3D-printing guides needle biopsies

The final models were printed in polylactic acid (PLA) using a fused deposition modeling (FDM) printer (i3 MK3S, Prusa Research, Prague, CZ) and a layer height of 0.3 mm.

**Figure 5:**
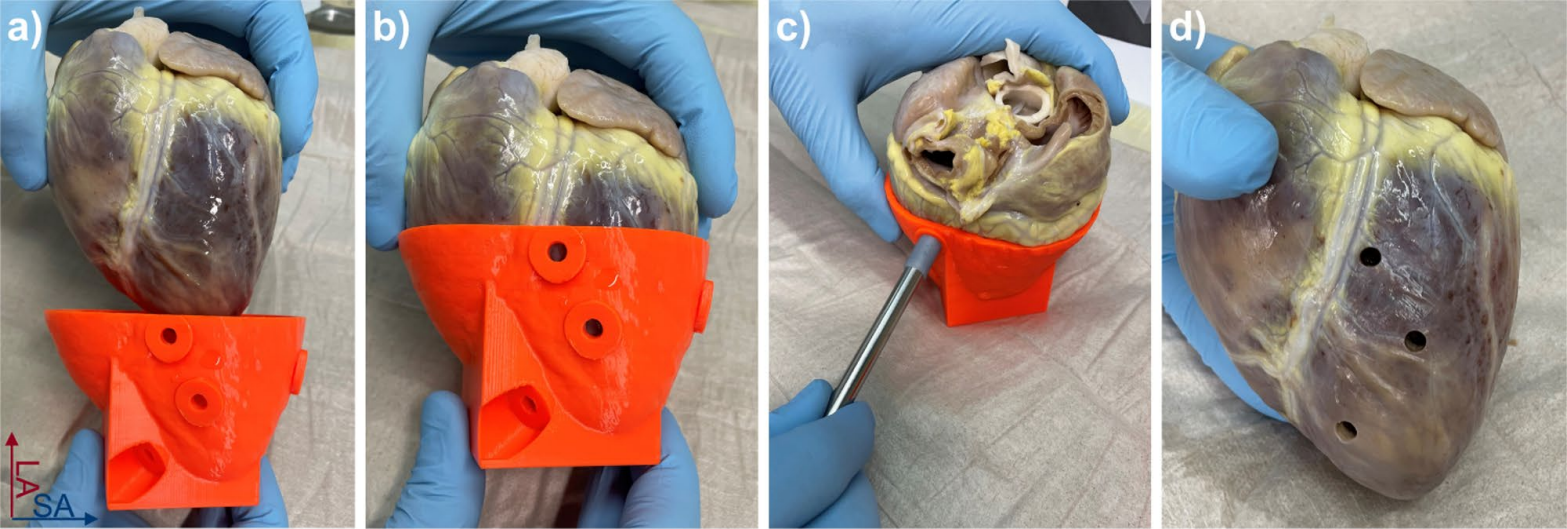
Photographs of the 3D printed guide and the corresponding heart (**a**). The heart takes a fixed position within the guide (**b**). The samples are extracted using a custom-made steel punch with variable insertion length (**c**). The insertion length can be varied by using different 3D-printed shims (**d**).

**Figure 6:**
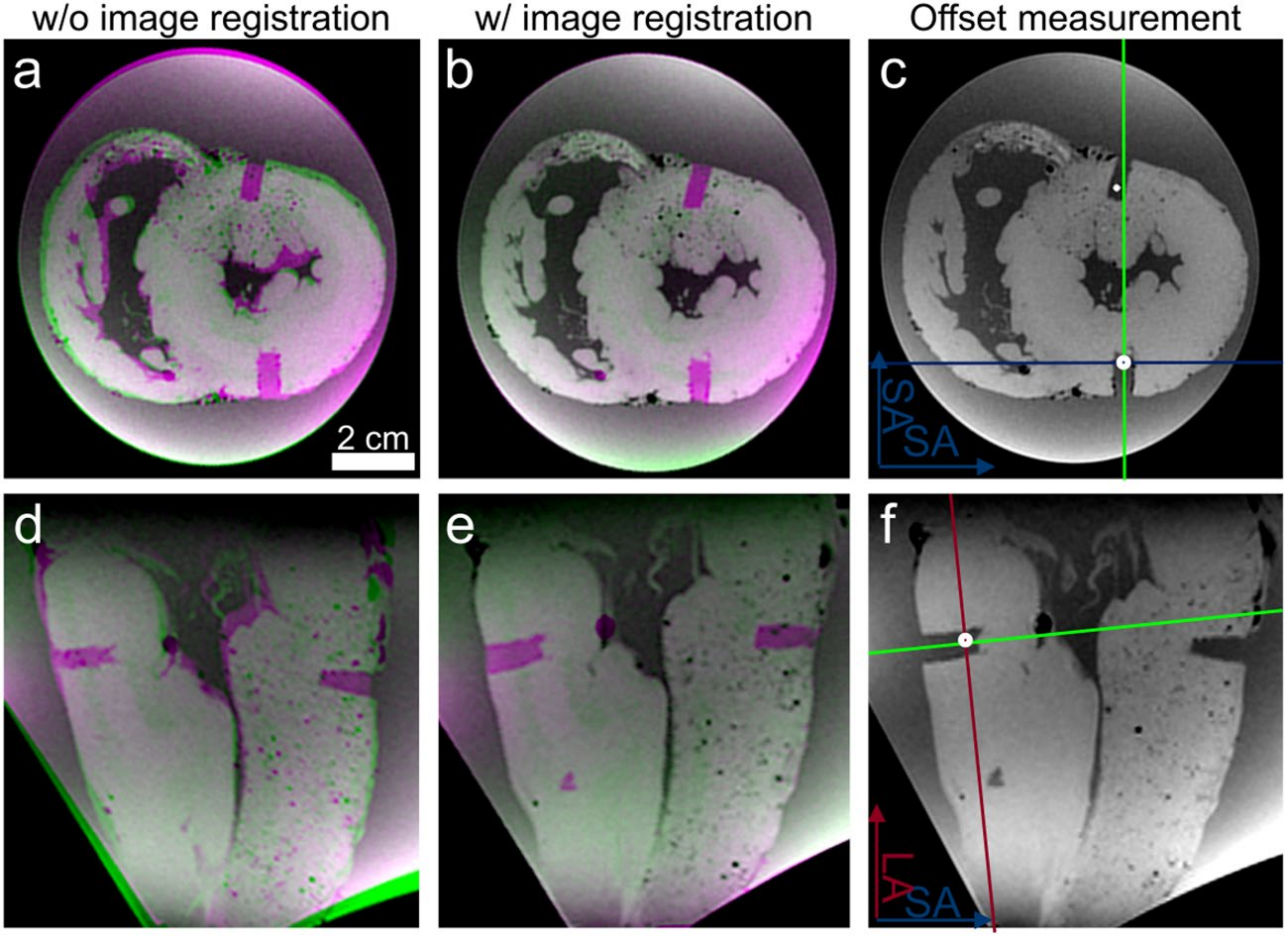
Example of co-registration of the 3D multi-echo datasets acquired before and after extraction of tissue samples: (**a**,**d**) without and (**b**,**e**) with co-registration. The target points are then overlaid on the dataset acquire after extraction of the samples to assess the precision of the sample extraction. The offset was measured with respect to the center line of the extracted cylindrical sample (green line) along both the short axis (blue line, **c**) and the long axis (red line, **f**).

A custom-made cylindrical biopsy punch with 5 mm inner diameter and a maximum depth of 20 mm was used for the extraction of the tissue samples (Fig. 4e). The maximum insertion depth of the punch can be adjusted using specific 3D shims. The shims were printed using a masked stereolithography (MSLA) printer (SL1, Prusa Research, Prague, CZ) for higher spatial resolution. To extract the samples, the punch was inserted 2 mm further than the calculated depth using the 3D printed shims to ensure the target point was captured and at a well-defined position along the axis of the extracted cylindrical sample. After extraction of the samples (Fig. 5), the hearts were imaged once again in the holder using the 3D multi-echo GRE sequence. The pre- and post-extraction datasets were co-registered with a 3D affine transformation and mean square error metric (MatLab function *imregtform*). For co-registration the segmented images were used after signal intensity and R2* thresholding to suppress the background and fixative. Potential deformations of the hearts after sample extraction were assessed by calculating the scaling factors in short and long axis direction from the transformation matrices that resulted from the co-registration. In addition, the accuracy of the co-registration was assessed by calculating the 3D normalized cross-correlation (NCC) between the pre- and post-extraction datasets both for the non-registered and the co-registered case. The NCC was calculated in MatLab^15^ and the maximum value of the 3D NCC matrix was used the measure for the accuracy of the co-registration. To assess the sample extraction precision the target points were superimposed on the post-extraction images and the lateral offset of the center of the extracted cylindrical tissue sample relative to the target point was measured (Fig. 6). In total, two distances were measured: the offsets in short axis and long axis direction of the heart. The mean of both offsets over all extracted samples were then tested for the hypothesis of being zero using a two-sided one-sample t-test. The absolute offset was tested for linear dependence on the target depths measured from the epicardial surface. Statistical significance was assumed for *p* < 0.05.

### Histology

For histology, tissue samples were fixed with 4% paraformaldehyde in PBS solution then rehydrated in 30% sucrose solution for 24-h. After gentle cleaning with isotonic saline, tissue was embedded in silicone molds and covered with OCT compound for cryopreservation at − 20 °C. Using a cryostat, 6 µm thick sections were collected every 120 µm for hematoxylin-staining with a 25% hematoxylin solution for 50 s. Further washing steps with saline were held to a minimum to avoid loss of MPIO binding. The primary objective of the staining was to enhance the visibility of background tissue structures to aid in the enumeration of MPIO. For analysis, a total of 3 slides covering the regions near the epicardial layer, the mid myocardium and the subendocardial layer were analyzed with brightfield microscopy using 100 × magnification.

## Results

### Myocardial tissue samples can be extracted with submillimeter precision

The acquisition time of the 3D multi-echo GRE sequence was 35.4 min per heart. Using the image data from this acquisition, 3D-printed guides could be successfully produced for all seven hearts. The creation of the digital models was fully automatic and required no manual input except the definition of the target locations. Printing time was $\lesssim$ 7.5 h per heart. Each 3D-printed guide fits the corresponding heart with no substantial clearance. Different operators could place the heart inside the holder reproducibly by sliding it inside the holder whereby the heart took its designated position. In total, 61 tissue samples were extracted from the seven hearts. The results of the scaling factors of the image co-registration show that the endocardial surface after sample extraction was larger in short axis by (0.31 ± 0.25)% and smaller in long axis direction by (1.65 ± 1.44)% (Supplementary Fig. S2). The mean of the NCC between the pre- and post-extraction image datasets was 0.905 ± 0.032 without and 0.982 ± 0.004 with co-registration (Supplementary Fig. S3).

### Ex vivo MRI shows excellent spatial correlation histology

**Figure 7:**
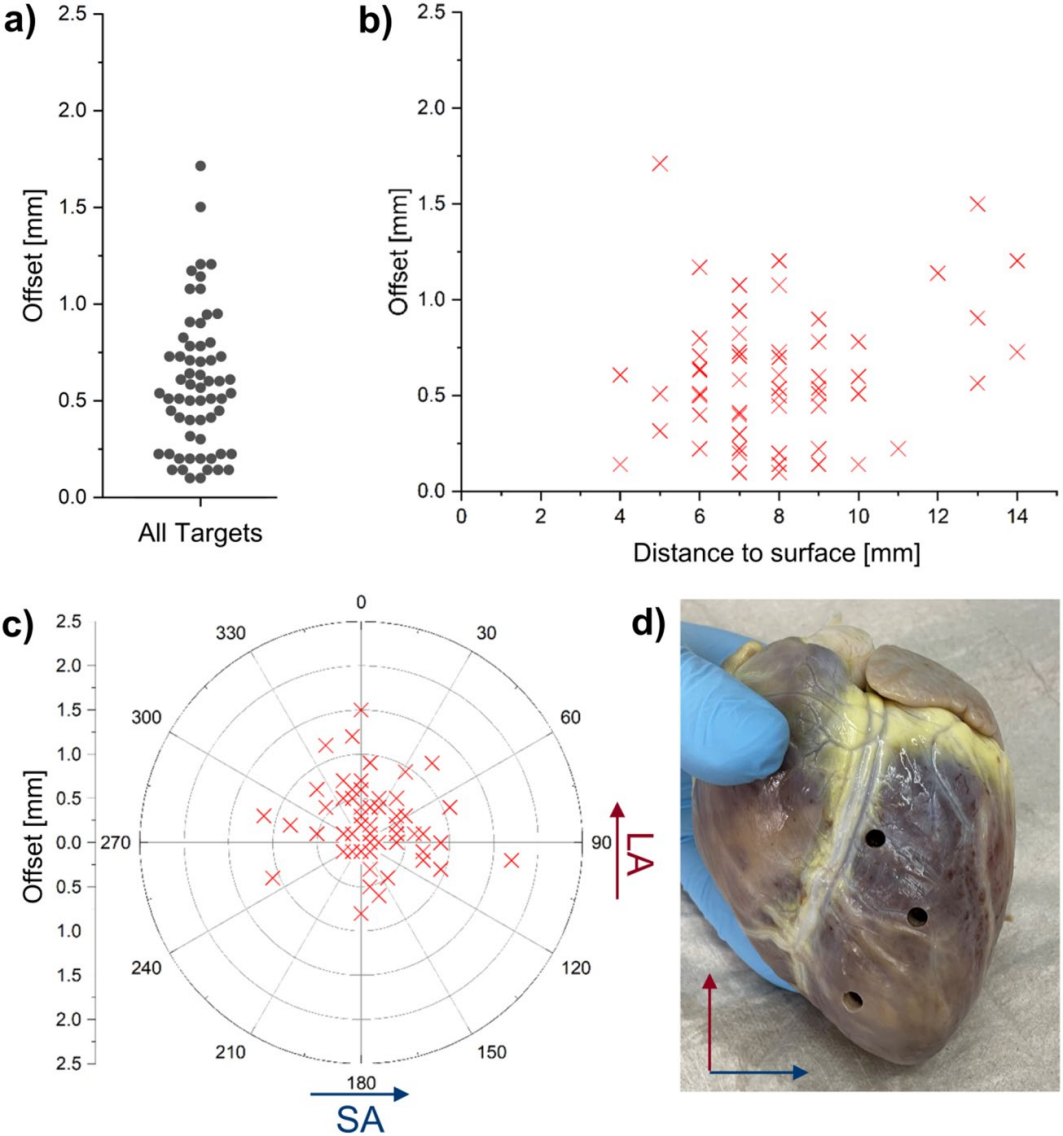
Results of the offsets measured for all 61 extracted samples from 7 different hearts. (**a**) Absolute values of the offsets. (**b**) Offsets plotted as a function of the target depth measured from the epicardial surface. (**c**) Polar plot of the offsets relative to the center line. (**d**) The offset is measured in two components along the short and long axes of the heart.

The absolute offsets of the center of the extracted sample from the target location were all ≤ 1.7 mm with the average being (0.59 ± 0.36) mm (Fig. 7a). In total, 53 out of all 61 samples (87%) had an offset of 1 mm or less. Thus, every target point was within the extracted sample (diameter: 5 mm). There was no significant linear dependence of the absolute offset on the distance of the target point to the epicardial surface (*p* = 0.11, Fig. 7b). Figure 7c shows a polar plot of the offsets with respect to the short and long axes of the heart, which are depicted in Fig. 7d. The mean of the offset along the short axis was (0.11 ± 0.48) mm and not significantly different from zero (*p* = 0.07). The mean offset along the long axis was (0.22 ± 0.43) mm being significantly larger than zero (*p* < 0.001).

**Figure 8:**
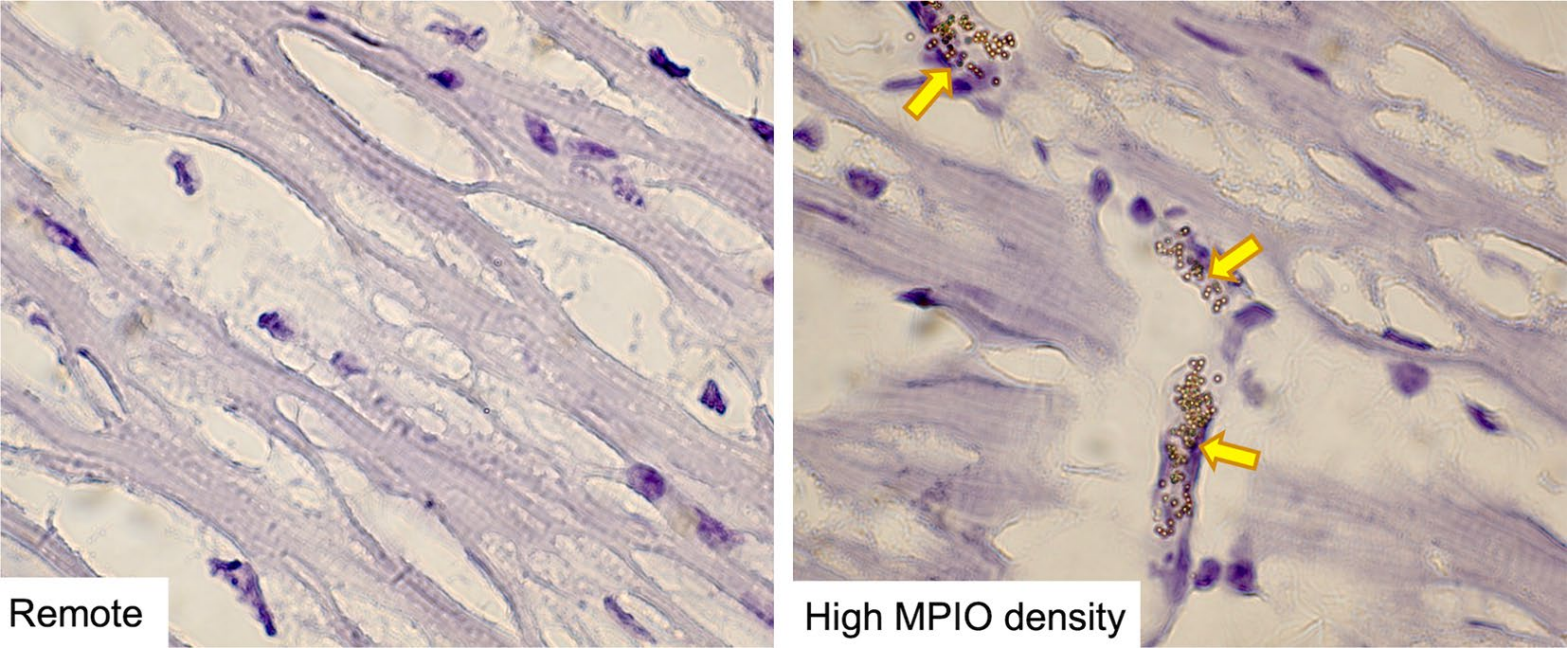
Ex vivo cryo-embedded histologic analysis stained with haematoxilin. The left image shows the histologic section of remote myocardium. On the contrary the right image shows the target area with high MPIO density (yellow arrows), which was manually selected using the R2* maps. Tissue was analyzed by using a ZEISS Axio Imager 2 optical microscope on 100 × magnification. Window levels were adapted to support the detection of MPIO.

The analysis of the histology showed, that no MPIOs were present in all samples taken from myocardial segments with no elevated R2*. Respectively, MPIOs were only seen in samples taken from segments with elevated R2* values. Figure 8 shows exemplary histology slices of two tissue samples that were extracted from a remote location and a location with hyperintense signal in the R2* images. As expected, no MPIOs are seen in the sample taken form the remote area whereas a high MPIO density is seen in the other sample.

## Discussion

In this study, a novel method is presented for a precise extraction of tissue samples from myocardial regions defined on high resolution ex vivo MRI. The method relies on the rigidity of the explanted hearts by using the unique shape of the epicardial surface to define a reference frame for the biopsy guides.

Here, a 3D model of the epicardial surface was created by R2* thresholding of the ex vivo image dataset. This step would not be necessary if the hearts were measured without being embedded in fixative as a signal intensity threshold could then be used. However, embedding the hearts allows for long scan times without potential tissue degradation so that the full potential of ex vivo imaging can be utilized. The large difference between R2* values of the myocardium and the fixative allows the use of thresholding for the segmentation of the myocardium. The 3D-printed shells fit the shape of the hearts such that the hearts are held in a fixed and reproducible position within the shell. The targeted tissue sample locations are extracted with an average precision of below 1 mm, with a maximum deviation of 1.7 mm. Considering that the size of the biopsy punch (here: 5 mm diameter) and the LV wall are typically substantially larger than that, the proposed technique provides a very high probability that the target region is contained within the extracted sample. Furthermore, the repetition of ex vivo imaging after tissue sample extraction and co-registration to the baseline images allows for a pixel-wise correlation of histology findings with ex vivo MRI results. So far, this has not been possible as typically gross short axis slices are taken from the heart, which need to be manually correlated with the MRI datasets. Correlation may be of particular value in studies of MI where the tissue appears heterogeneous, e.g., at the border zones around the infarcted core, which extend over less than 1 mm^6^.

The precision measurement indicates that there is a small bias of 0.22 mm in long axis direction of the heart, i.e. in the vertical direction of the 3D printed guide. Thus, the center of the extracted samples is shifted towards the apical direction of the heart. This could be a result of the printing technique and might be specific to a certain type of 3D printer. However, also a slight deformation of the hearts was seen after sample extraction. Even though the deformation is only of the order of one percent, a larger deformation of the shape along the long axis was found compared to the short axis. The length of the long axis of the ventricles was about 80 mm, i.e. the distance of the base and apex to the center was 40 mm. The average scaling factor in long axis direction of 1.65% thus leads to an absolute difference of 0.66 mm at the base and apex, respectively. As most of the samples were taken at the mid-ventricular level, where the absolute scaling was less than 0.66 mm, the deformation may be a possible explanation of the measured offset of the extracted samples towards the apex. The pressure being applied to the heart during the sample extraction process to hold the hearts in the 3D-printed holders should thus be kept minimal. A possible solution would be to slightly enlarge the holders along the long axis and thus reduce inward pressure on the apex. Furthermore, the holders could be printed more precisely by a reduction of layer height, albeit at the expense of increased printing times.

In this study, no samples were extracted from the septal wall. However, the presented technique may also be suitable to extract samples from this region by using longer biopsy needles of about 3 cm to cover the distance from the right ventricular wall to the septum. As an alternative, the right ventricle could be cut away before creation of the 3D model and the biopsy guide. This would provide direct access to the septal wall while maintaining a convex surface. In general, the proposed method can be adapted to any needle/punch diameter and shape. The precision of the 3D printing may however be a limitation for very thin needles in terms of the precision relative to the needle diameter. In addition, needles/punches with arbitrary length can be used; however, the precision may be impaired for target points that are very distant from the epicardial surface.

An important aspect of ex vivo MRI heart studies is connecting the results with in vivo findings. However, a limitation of the proposed technique in its current form is the lack of correlation between histology findings, ex vivo and an in vivo MRI. A previously published technique uses 3D-printed scaffolds to shape the extracted hearts similar to the in vivo geometry^16^. This can be applied to spatially correlate ex vivo MRI to the in vivo image data. Combining this with the proposed technique for the extraction of biopsy samples, histology findings could be connected to both ex vivo and in vivo MRI. Another limitation is that the method is time consuming due to the waiting time after fixation of the hearts, the long imaging time for the high resolution multi-echo GRE dataset as well as time for 3D printing (Fig. 1). The waiting time for the fixative to diffuse through the myocardium could be substantially reduced by perfusion the heart with the fixative after extraction. Furthermore, both the MR imaging and 3D printing time could be reduced by decreasing the spatial resolution. However, further studies are necessary to assess whether this would impair the precision of the sample extraction process. A further limitation is that the method has not been tested for the extraction of samples from the atria or right ventricle. The method relies on the rigidity of the myocardium, particularly during introduction of the biopsy punch. Both the atria and right ventricle are substantially thinner than the myocardium and thus less rigid. To overcome this, a scaffold for the endocardial walls could be printed similar to the previously published technique mentioned above^16^ that supports the right ventricle or atria when the sample is extracted. Another limitation is the loss of rotational information of the extracted tissue sample, which is of importance particularly in the context of diffusion tensor imaging. However, retaining the rotational orientation could be achieved for example, by adding a notch to the biopsy punch as a reference angle or by using an asymmetric punch shape.

Computed tomography (CT) would be a possible alternative to MRI to acquire high-resolution images of explanted hearts for the planning ex vivo biopsies. However, as the MPIOs have a size of only one micrometer they cannot be visualized with clinical CT scanners. Even if several MPIOs agglomerate, the size is below the resolution of CT scanners which is at the order of 250 µm. Thus, CT is not suitable for guiding biopsies in studies that use MPIO-based molecular contrast agents.

In conclusion, the proposed technique provides an automated framework to create 3D printed guides for the precise extraction of myocardial tissue samples. This allows for a direct spatial correlation of histology findings to ex vivo MRI. The technique may be adapted to ex vivo studies of other rigid organs and extraction instruments like biopsy needles.

### Supplementary Information

Supplementary Figures.

## Data Availability

The datasets used and/or analyzed during the current study are available from the corresponding author on reasonable request.
