# Supplementary material for: Combination of high resolution MRI with 3D-printed needle guides for ex vivo myocardial biopsies
Source: Sci Rep. 2024 Jan 5;14:606. doi: 10.1038/s41598-023-50943-2 (PMC10770147; doi:10.1038/s41598-023-50943-2)
Supplement: Supplementary file 1 — Supplementary Figures. [file 41598_2023_50943_MOESM1_ESM.docx]

**Supplementary Figures**


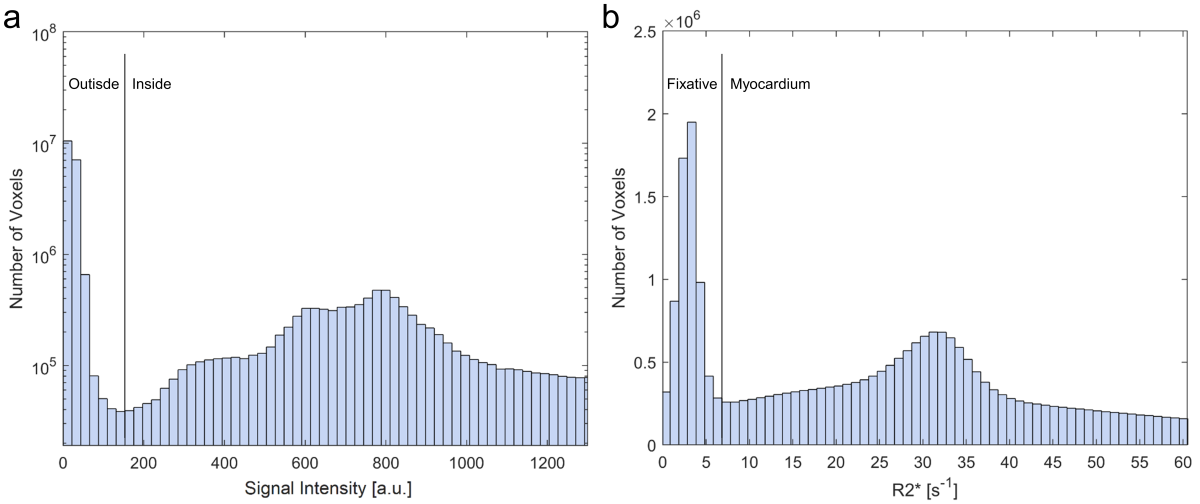


**Supplementary Figure 1.** Exemplary histograms of the signal intensity (a) and R2* values (b) of an ex vivo measurement. The threshold for the signal intensity was set manually for each heart (here: 175) to exclude the region outside the container, i.e. air. The threshold for R2* was set to 10s^-1^ for all hearts to suppress the fixative, which has a mean of (31.2 ± 3.5) s^-1^ showing no substantial variation between different hearts.


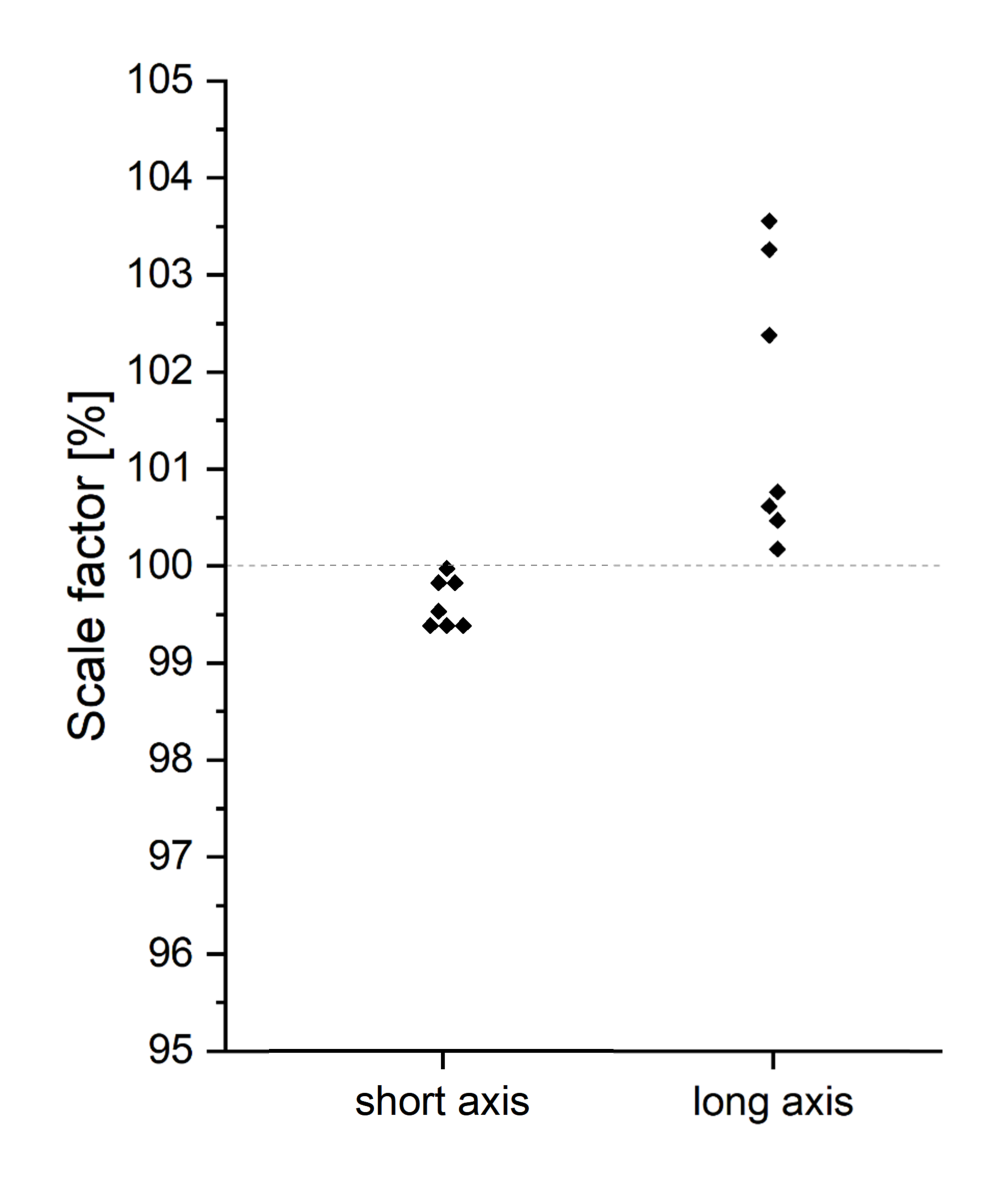


**Supplementary Figure 2.** Results of the scaling factors used to co-register the post- to the pre-extraction images of the hearts. Here, a scale factor of below 100 % is indicative of the heart being larger in the post-extraction dataset. Thus, the values show that the hearts were larger in short axis and smaller in long axis direction post sample extraction.


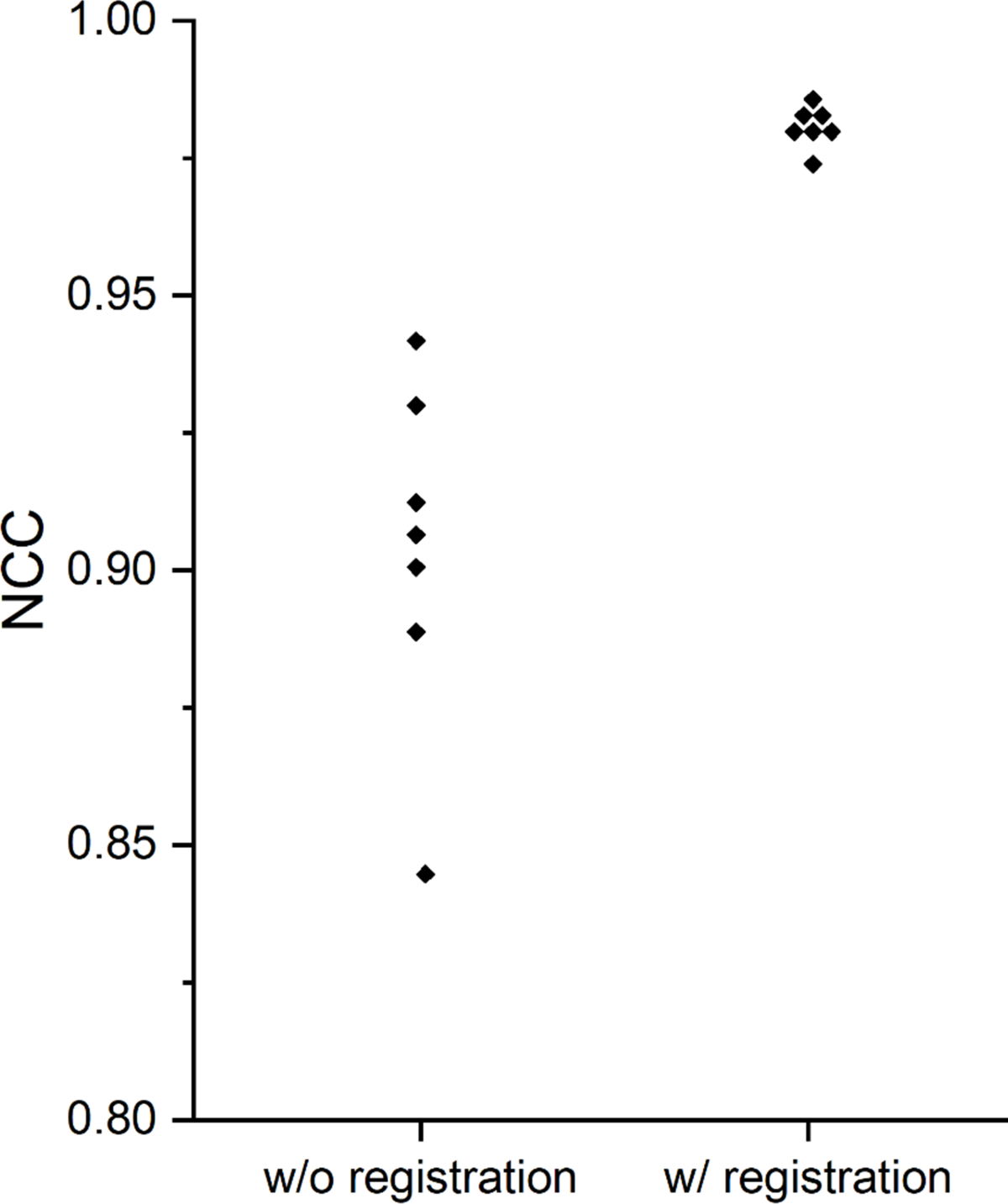


**Supplementary Figure 3.** Values of the normalized cross-correlation (NCC) between the pre- and post-extraction datasets. The NCC is calculated for all seven hearts without and with co-registration.
